# Spironolactone treatment attenuates vascular dysfunction in type 2 diabetic mice by decreasing oxidative stress and restoring NO/GC signaling

**DOI:** 10.3389/fphys.2015.00269

**Published:** 2015-10-05

**Authors:** Marcondes A. B. Silva, Thiago Bruder-Nascimento, Stefany B. A. Cau, Rheure A. M. Lopes, Fabiola L. A. C. Mestriner, Rafael S. Fais, Rhian M. Touyz, Rita C. Tostes

**Affiliations:** ^1^Department of Pharmacology, Ribeirao Preto Medical School, University of Sao PauloRibeirão Preto, Brazil; ^2^BHF Glasgow Cardiovascular Research Centre, Institute of Cardiovascular and Medical sciences, University of GlasgowGlasgow, UK

**Keywords:** type 2 diabetes, aldosterone, mineralocorticoid receptor, vascular, oxidative stress

## Abstract

Type 2 diabetes (DM2) increases the risk of cardiovascular disease. Aldosterone, which has pro-oxidative and pro-inflammatory effects in the cardiovascular system, is positively regulated in DM2. We assessed whether blockade of mineralocorticoid receptors (MR) with spironolactone decreases reactive oxygen species (ROS)-associated vascular dysfunction and improves vascular nitric oxide (NO) signaling in diabetes. Leptin receptor knockout [LepR^db^/LepR^db^ (db/db)] mice, a model of DM2, and their counterpart controls [LepR^db^/LepR^+^, (db/+) mice] received spironolactone (50 mg/kg body weight/day) or vehicle (ethanol 1%) via oral per gavage for 6 weeks. Spironolactone treatment abolished endothelial dysfunction and increased endothelial nitric oxide synthase (eNOS) phosphorylation (Ser^1177^) in arteries from db/db mice, determined by acetylcholine-induced relaxation and Western Blot analysis, respectively. MR antagonist therapy also abrogated augmented ROS-generation in aorta from diabetic mice, determined by lucigenin luminescence assay. Spironolactone treatment increased superoxide dismutase-1 and catalase expression, improved sodium nitroprusside and BAY 41-2272-induced relaxation, and increased soluble guanylyl cyclase (sGC) β subunit expression in arteries from db/db mice. Our results demonstrate that spironolactone decreases diabetes-associated vascular oxidative stress and prevents vascular dysfunction through processes involving increased expression of antioxidant enzymes and sGC. These findings further elucidate redox-sensitive mechanisms whereby spironolactone protects against vascular injury in diabetes.

## Introduction

In the past two decades, the number of people diagnosed with diabetes worldwide has dramatically increased (Zimmet et al., 2001). Currently, type 2 diabetes mellitus (DM2), characterized by insulin resistance and/or abnormal insulin secretion, either of which may predominate, affects 7.6 million people in Brazil, 26 million in the United States and 347 million worldwide (Zimmet et al., 2001; Manrique et al., 2014).

DM2 is an important cause of cardiovascular and renal mortality and morbidity. Oxidative stress, defined as increased bioavailability of reactive oxygen species (ROS) (Danaei et al., 2011), resulting from enhanced oxidative activity or decreased antioxidant capacity, is typically described in DM2, and is associated with vascular damage. We have recently shown that db/db mice, a DM2 experimental model, present increased expression/activity of nicotinamide adenine dinucleotide phosphate (NADPH) oxidase (Nox) isoforms leading to inflammation and, consequently, to vascular dysfunction (Bruder-Nascimento et al., 2015). On the other hand, reduced antioxidant enzymes expression/activity has also been reported in DM2 (Bruder-Nascimento et al., 2014).

One of the many mechanisms associated with oxidative stress-induced vascular dysfunction is a decrease in nitric oxide (NO) bioavailability. Increased ROS production impairs NO effects (Förstermann and Münzel, 2006; Fels et al., 2010; Stasch et al., 2011; Velmurugan et al., 2013), especially NO-induced activation of soluble guanylyl cyclase (sGC), cyclic guanosine monophosphate (cGMP) production, and smooth muscle cells relaxation (Förstermann and Münzel, 2006; Fels et al., 2010; Stasch et al., 2011). In genetic and high-fat diet-induced DM2 experimental models, increased generation of superoxide anion (O${}_{2}^{-}$), a ROS that avidly reacts with vascular NO to form peroxynitrite (ONOO^−^), has been consistently reported (Hink et al., 2001; Förstermann and Münzel, 2006; Bruder-Nascimento et al., 2014). Molecular mechanisms and factors that contribute to DM2-associated vascular dysfunction are not yet completely understood.

Preclinical and clinical studies have shown that aldosterone is positively regulated in DM2 (Pieper, 1997; Heitzer et al., 2000; Bruder-Nascimento et al., 2014). Aldosterone, a steroid hormone from the mineralocorticoid family, is critically involved in electrolyte balance and blood pressure control. Aldosterone actions are mediated mainly through mineralocorticoid receptors (MR) via well-characterized genomic mechanisms. In addition to long-term transcriptional effects, aldosterone has important acute or non-genomic actions in the cardiovascular system, influencing vascular reactivity and remodeling, endothelial function, inflammatory, and redox processes (Hollenberg et al., 2004; Touyz, 2004; Callera et al., 2011; Briones et al., 2012; Bruder-Nascimento et al., 2014).

A recent study showed that diabetic individuals treated with a MR antagonist have improved coronary microvascular function, raising the possibility that MR blockade is beneficial and may prevent cardiovascular disease in patients with DM2 (Toda et al., 2013). This important finding supports the notion that dysregulated aldosterone production contributes to the development and progression of cardiovascular disease in DM2. Mechanisms involved in these beneficial effects of MR blockade remain unclear.

Considering that aldosterone has pro-oxidative, pro-growth and pro-inflammatory effects, which promote vascular damage (Callera et al., 2011; Nguyen Dinh Cat and Jaisser, 2012; Ronconi et al., 2012; Toda et al., 2013), and that aldosterone levels are increased in DM2 (Hollenberg et al., 2004; Briones et al., 2012), we assessed whether spironolactone, a MR antagonist, decreases ROS-associated vascular dysfunction and improves vascular NO signaling in db/db mice. We tested the hypothesis that spironolactone augments NO/cGMP-mediated vascular responses in DM2, through redox-sensitive processes.

## Materials and methods

### Animals and spironolactone treatment

All experimental procedures were approved by the Ethics Committee on Animal Research of the Ribeirao Preto Medical School, University of Sao Paulo (protocol n° 062/2012) and are in accordance with the Guidelines of the Brazilian College of Animal Experimentation (COBEA).

Fourteen male, 12–14 weeks-old, [B6.BKS(D)-Lepr < db>/J] mice (db/db), an experimental model of DM2, and 14 age-matched heterozygous nondiabetic mice [(db/+), from The Jackson Laboratory, Maine, USA were used in the study. db/+ and db/db mice were divided into four groups (7 mice per group): db/+ spironolactone and db/db spironolactone, which were treated for 6 weeks with the MR antagonist spironolactone (50 mg/kg body weight/day), and db/+ vehicle and db/db vehicle, which were treated with ethanol 1% via oral per gavage (0.1 ml per 10 g of the total body weight). The oral gavage procedure was performed once a day and always at the same time of the day (6:30 to 7:00 p.m.). Twelve hours after the last dose, mice were used for the experimental protocols. The animals were anesthetized (2% isoflurane vaporized with oxygen), killed by cervical dislocation and tissues of interest were collected.

Mice were kept in the animal facility of the Department of Pharmacology, Ribeirao Preto Medical School, University of Sao Paulo, under controlled temperature (22–24°C) and humidity, 12-h light/dark cycles, fed with standard diet and water *ad libitum*.

### Metabolic profile

Serum sodium (Na^+^) and potassium (K^+^) were determined using ion selective electrodes by potentiometry in an automatic biochemistry analyzer (BT 3000 plus, Wiener Lab, Rosario, Argentina). Serum aldosterone and insulin was determined by enzyme immunoassay (Aldosterone Rodent ELISA Kit, Abnova, Taipei, Taiwan/Rat-Mouse Insulin ELISA, Millipore, Darmstadt, Germany). Blood glucose levels were measured with a portable glucose meter (Accu-Chek Active®, Roche Diagnostics), before treatment was started (week 0), at the third week (week 3), and at the end of treatment (week 6), after 12-h fasting, in all experimental groups.

### Systolic blood pressure measurement

Systolic blood pressure (SBP) was assessed by a tail-cuff pletysmography (CODA™ High Throughput - Kent Scientific Corporation) before treatment was started (week 0) and at the end of the spironolactone/vehicle treatment (week 6).

### Vascular function

Mesenteric vascular beds were isolated from db/db and db/+ mice treated with vehicle or spironolactone. Second-order branches of superior mesenteric artery were dissected and mounted on a wire myograph (DMT, Danish Myo Technology, Aarhus, Denmark). Vessel segments (2 mm in length) were mounted on 25 μm wires in a vessel bath chamber for isometric tension recording and equilibrated for 30 min in Krebs–Henseleit-modified physiological salt solution (120 mmol/L NaCl, 25 mmol/L NaHCO_3_, 4.7 mmol/L KCl, 1.18 mmol/L KH_2_PO_4_, 1.18 mmol/L MgSO_4_, 2.5 mmol/L CaCl_2_, 0.026 mmol/L EDTA, and 5.5 mmol/L glucose), at 37°C, continuously bubbled with 95% O_2_ and 5% CO_2_, pH 7.4. At the beginning of each experiment, arteries were contracted with 10 μmol/L noradrenaline (NE) to test for functional integrity. In some experiments, the endothelium was removed by gently rubbing the luminal side of the vascular segments. The integrity of the endothelium or its removal was assessed by the presence or absence of relaxation, respectively, of NE pre-contracted arteries in response to 1 μmol/L acetylcholine (Ach).

Concentration–response curves to Ach (10^−10^–10^−5^ M) were performed in endothelium-intact arteries to assess endothelium-dependent relaxation. Concentration-response curves to sodium nitroprusside (SNP, 10^−10^–10^−5^ M), BAY 41-2272 (10^−10^–10^−5^ M) and 8-Br-cGMP (10^−8^–10^−4^ M) were performed in endothelium-denuded arteries. In some protocols, arteries were pre-incubated with Nitro- L -arginine methyl ester hydrochloride [(L-NAME) 10^−4^ M] or tempol (10^−4^ M), 30 min prior to the concentration-response curves.

### Lucigenin-enhanced chemiluminescence

Vascular ROS generation was measured by a luminescence assay using lucigenin as the electron acceptor and NADPH as the substrate. Aortic rings from db/db and db/+ mice treated with vehicle or spironolactone were homogenized in assay buffer (50 mmol/L KH_2_PO_4_, 1 mmol/L EGTA and 150 mmol/L sucrose, pH 7.4) with a glass-to-glass homogenizer. The assay was performed with 100 μL of sample, 1.25 μL of lucigenin (5 μmol/l), 25 μL of NADPH (0.1 mmol/l) and assay buffer to a total volume of 250 μL. Luminescence was measured for 30 cycles of 18 s each by a luminometer (Lumistar Galaxy, BMG Labtechnologies, Ortenberg, Germany). Basal readings were obtained prior to the addition of NADPH and the reaction was started by the addition of the substrate. Basal and buffer blank values were subtracted from the NADPH-derived luminescence. Superoxide production was expressed as relative luminescence unit (RLU)/μg of protein.

### Western blotting

Total protein was extracted from mesentery beds. Frozen tissues were homogenized in 50 mmol/L Tris/HCl (pH 7.4) lysis buffer (containing 1% Nonidet P-40, 0.5% sodium deoxycholate, 150 mmol/L NaCl, 1 mmol/L EDTA, 0.1% SDS, 2 mmol/L Na_3_VO_4_, 1 mmol/L PMSF, 1 μg/mL pepstatin A, 1 μg/mL leupeptin, and 1 μg/mL aprotinin). Total protein extracts were cleared by centrifugation at 12,000 ***g*** for 10 min and the pellet was discarded. Proteins from homogenates of vascular tissues (50 μg) were separated by electrophoresis on a polyacrylamide gel (10%) and transferred on to a nitrocellulose membrane. Non-specific binding sites were blocked with 5% skim milk or 1% BSA in Tris-buffered saline solution with Tween 1% at 24°C. Membranes were then incubated with specific antibodies overnight at 4°C. Antibodies were as follows: Ser^1177^- endothelial Nitric Oxide Synthase (eNOS) and total eNOS (Cell Signaling, 1:500), soluble guanylyl cyclase (sGC) α and β subunits (Abcam, 1:500), Superoxide Dismutase 1 (SOD1), SOD2 and Catalase. Antibody to β-actin (Sigma) was used as internal housekeeping control. After incubation with secondary antibodies, signals were revealed with chemiluminescence, visualized by autoradiography and quantified densitometrically.

### Data and statistical analyses

Mice were divided into 7 animals per group. Relaxation responses to Ach, SNP, BAY 41-2272 and 8-Br-cGMP are expressed as a percentage of contraction in response to NE. The individual concentration–response curves were fitted into a curve by non-linear regression analysis. p*D*_2_ (defined as the negative logarithm of the EC_50_ values) and maximal response (*E*_max_) values were analyzed. Lucigenin and western blot results were analyzed on the raw data and presented as percentage of the db/+ group, which was represented as 100%. Data are represented as mean ± Standard Error of the Mean (SEM) with a probability of *P* < 0.05 used for significance. All parameters and calculations were analyzed using Two-Way analysis of variance (ANOVA) followed by the Bonferroni′s *post-hoc* test. The Prism software, version 5.0 (GraphPad Software Inc., San Diego, CA, USA) was used to analyze the results.

## Results

### Effects of spironolactone treatment on systolic blood pressure, glucose levels, and biochemistry parameters

Db/db mice exhibited higher plasma glucose levels compared to db/+ mice (*p* < 0.05). No differences in SBP were observed among the groups. Spironolactone treatment did not change SBP or glucose levels, either in control or db/db mice (Table 1).

**Table 1:** **Fasting blood glucose and systolic blood pressure in db/db and db/+ mice during treatment with spironolactone or vehicle for 6 weeks**.

**Parameters and groups**	**Week 0**	**Week 3**	**Week 6**
**FASTING GLUCOSE LEVELS (mmol/L)**			
db/+ vehicle	5.71 ± 0.14	5.91 ± 0.42	6.15 ± 0.16
db/+ spironolactone	5.85 ± 0.23	6.09 ± 0.35	5.74 ± 0.43
db/db vehicle	14.89 ± 2.63^*^	14.58 ± 2.60^*^	14.20 ± 1.47^*^
db/db spironolactone	13.81 ± 2.40^*^	13.87 ± 1.40^*^	13.60 ± 0.91^*^
**SYSTOLIC BLOOD PRESSURE (mmHg)**			
db/+ vehicle	135.64 ± 3.88	–	131.86 ± 2.73
db/+ spironolactone	133.55 ± 2.15	–	130.30 ± 2.52
db/db vehicle	139.74 ± 5.40	–	132.94 ± 3.16
db/db spironolactone	142.74 ± 6.91	–	130.10 ± 4.08

*Data are expressed as mean ± SEM*.

* *P < 0.05 vs. respective db/+ group; n = 6–7*.

**Figure 1:**
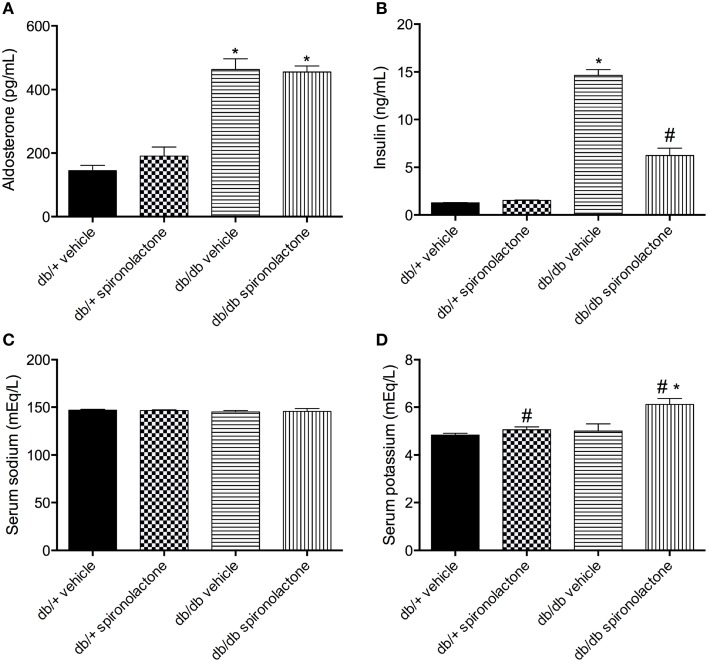
**Serum levels of aldosterone (A), insulin (B), sodium (C) and potassium (D) in db/db and db/+ mice treated with vehicle or spironolactone for 6 weeks**. Data are expressed as mean ± SEM. ^*^P < 0.05 vs. respective db/+ group, ^#^*P* < 0.05 vs. respective vehicle-treated group; *n* = 6–7.

Db/db mice displayed increased aldosterone and insulin levels compared to non-diabetic db/+ mice. Spironolactone treatment increased plasma K^+^ levels in both db/+ and db/db mice. In addition, the treatment reduced insulin levels in diabetic mice. No differences were observed in Na^+^ levels among the groups (Figure 1).

### Effect of spironolactone on endothelium-dependent relaxation in arteries from db/+ and db/db mice

**Figure 2:**
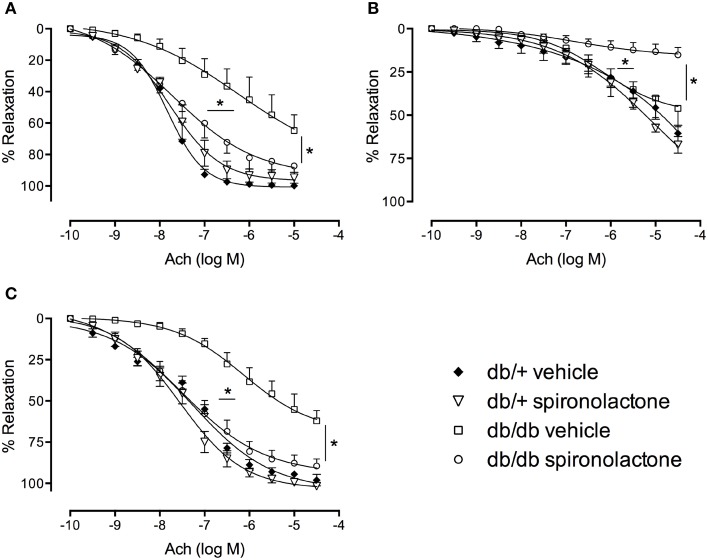
**Ach-induced vascular relaxation in mesenteric arteries from db/db and db/+ mice treated with spironolactone or vehicle for 6 weeks**. **(A)** Ach; **(B)** Ach + L-NAME 10^−4^ M; and **(C)** Ach + tempol 10^−4^ M. Arteries were incubated with L-NAME or tempol 30 min before performing Ach concentration-response curves. Data are expressed as mean ± SEM. ^*^P < 0.05 db/db vehicle vs. all other groups; *n* = 6.

Figure 2 shows the effects of spironolactone on vascular function of db/+ and db/db mice. Relaxation responses to Ach were significantly reduced in arteries from db/db mice, but not in arteries from db/db mice treated with the MR antagonist. The non-selective Nitric Oxide Synthase (NOS) inhibitor L-NAME attenuated Ach-induced vasorelaxation in all groups, but with minimal or no effects in arteries from db/db mice. L-NAME almost fully abolished Ach-induced relaxation in arteries from db/db mice treated with spironolactone.

Tempol did not modify Ach responses in any group, including arteries from db/db mice, suggesting that ^−^O_2_ does not contribute to the impaired Ach-induced relaxation in arteries from diabetic mice.

### Effect of spironolactone on endothelium-independent relaxation in arteries from db/+ and db/db mice

**Figure 3:**
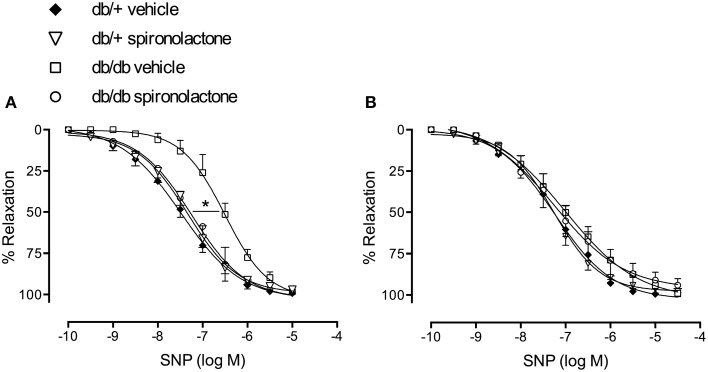
**SNP-induced vascular relaxation in mesenteric arteries from db/db and db/+ mice treated with spironolactone or vehicle for 6 weeks**. **(A)** SNP; **(B)** SNP + tempol 10^−4^ M. Arteries were incubated with tempol 30 min before performing SNP concentration-response curves. Data are expressed as mean ± SEM. ^*^P < 0.05 db/db vehicle vs. all other groups; *n* = 6.

Figure 3A illustrates the effects of spironolactone on endothelium-independent relaxation in mesenteric arteries from db/+ and db/db mice. Endothelium-denuded arteries from diabetic mice presented impaired relaxant responses to SNP, characterized by a right-shift in the concentration-response curves, vs. arteries from non-diabetic mice. Spironolactone treatment abrogated this dysfunction. Unlike its lack of effects on Ach-induced relaxation, tempol restored relaxation responses to SNP in arteries from db/db mice and no differences were observed among the groups (Figure 3B).

**Figure 4:**
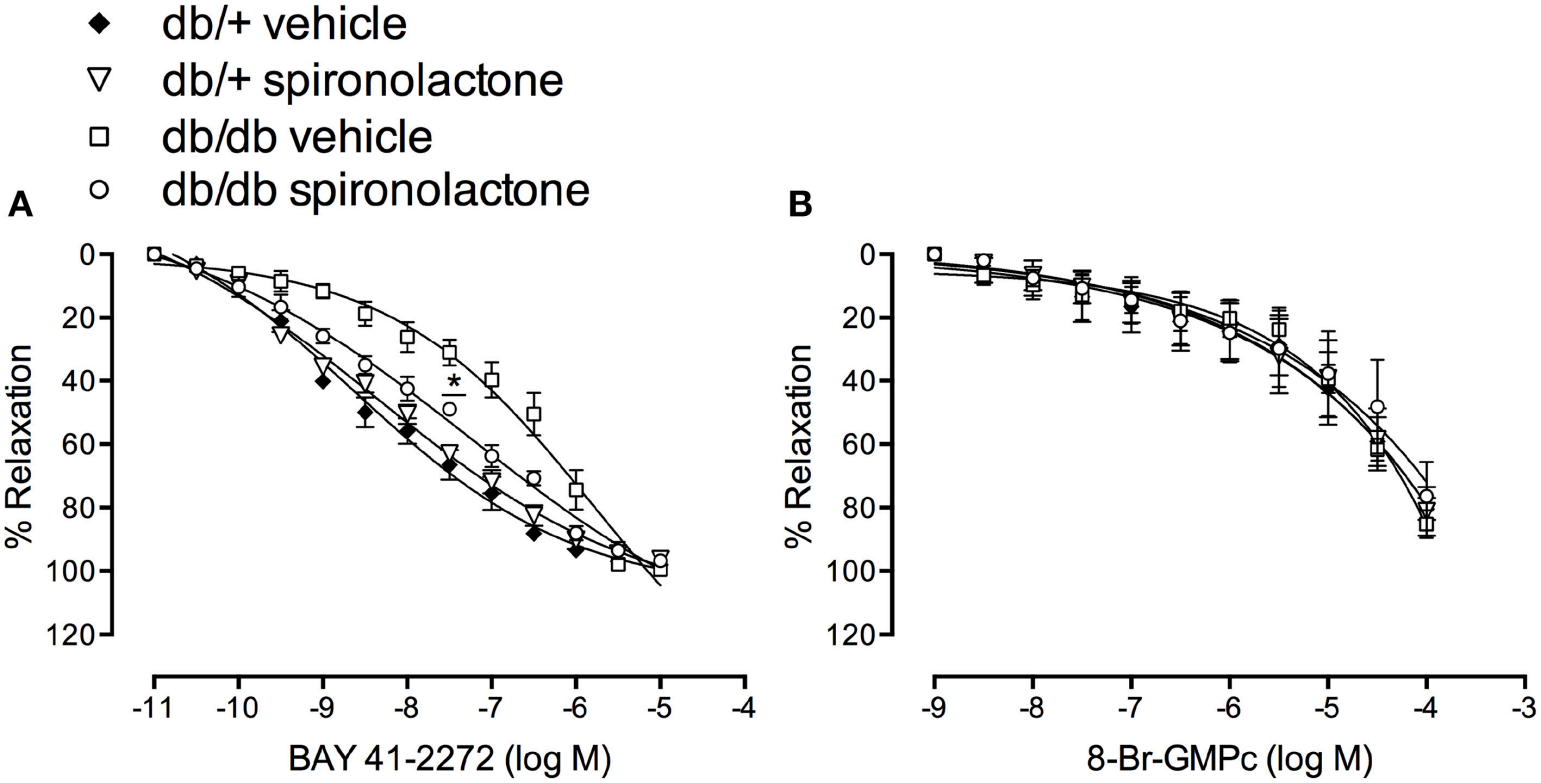
**Vascular relaxation induced by BAY 41-2272 (A) and 8-Br-cGMP (B) in mesenteric arteries from db/db and db/+ mice treated with spironolactone or vehicle for 6 weeks**. Data are expressed as mean ± SEM. ^*^P < 0.05 db/db vehicle vs. all other groups; *n* = 6.

To assess changes in sGC/cGMP signaling, we performed concentration-response curves to BAY 41-2272, a soluble sGC stimulator, and to the cGMP analog, 8-Br-cGMP. Endothelium-denuded arteries from db/db mice exhibited impaired relaxation to BAY 41-2272, represented by a right-shift in BAY 41-2272 responses. Spironolactone abrogated this abnormality in arteries from db/db mice (Figure 4A). No differences in cGMP-induced vasodilation were observed among the groups (Figure 4B).

### Expression of sGC and eNOS isoform in arteries from db/+ and db/db mice

**Figure 5:**
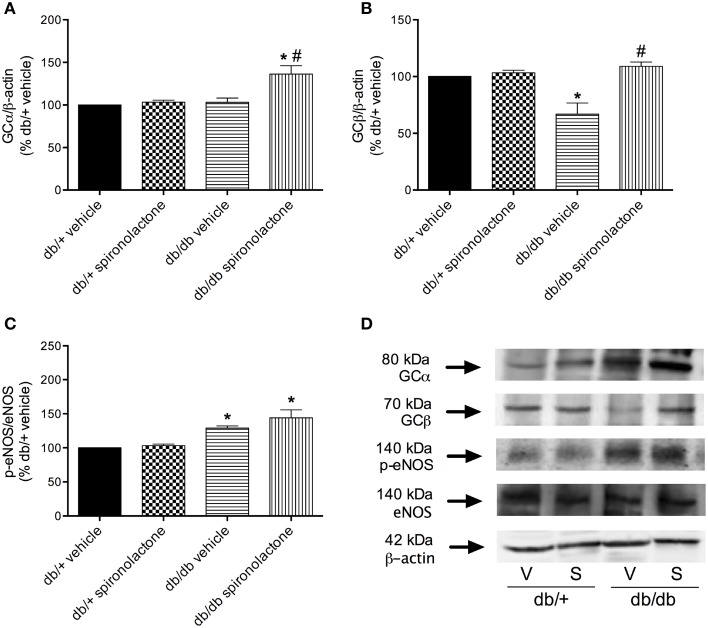
**Protein expression of sGC α (A), sGC β (B) and p-eNOS (C) protein in mesenteric arteries from db/db and db/+ mice treated with spironolactone or vehicle for 6 weeks**. Representative images are displayed in **(D)**. β-actin and total eNOS expression were also determined and used as internal controls. Data are expressed as mean ± SEM. ^*^P < 0.05 vs. respective db/+ group, ^#^*P* < 0.05 vs. db/db vehicle; V, vehicle; S, spironolactone, *n* = 5.

To determine whether impaired BAY 41-2272-induced relaxation in arteries from db/db mice was associated with downregulation of sGC, vascular sGC protein content was determined. Figures 5A,B show that mesenteric arteries from db/db and non-diabetic mice present similar expression of sGC α subunit. However, decreased sGC β subunit protein expression was observed in arteries from db/db mice compared to db/+ mice. Spironolactone treatment increased sGC α subunit and restored sGC β subunit expression in arteries from db/db mice.

Arteries from db/db mice treated with vehicle or spironolactone exhibited increased eNOS phosphorylation at Ser^1177^ compared to arteries from their respective vehicle- and spironolactone-treated control db/+ groups (Figures 5C,D).

### Redox status in arteries from db/+ and db/db mice

**Figure 6:**
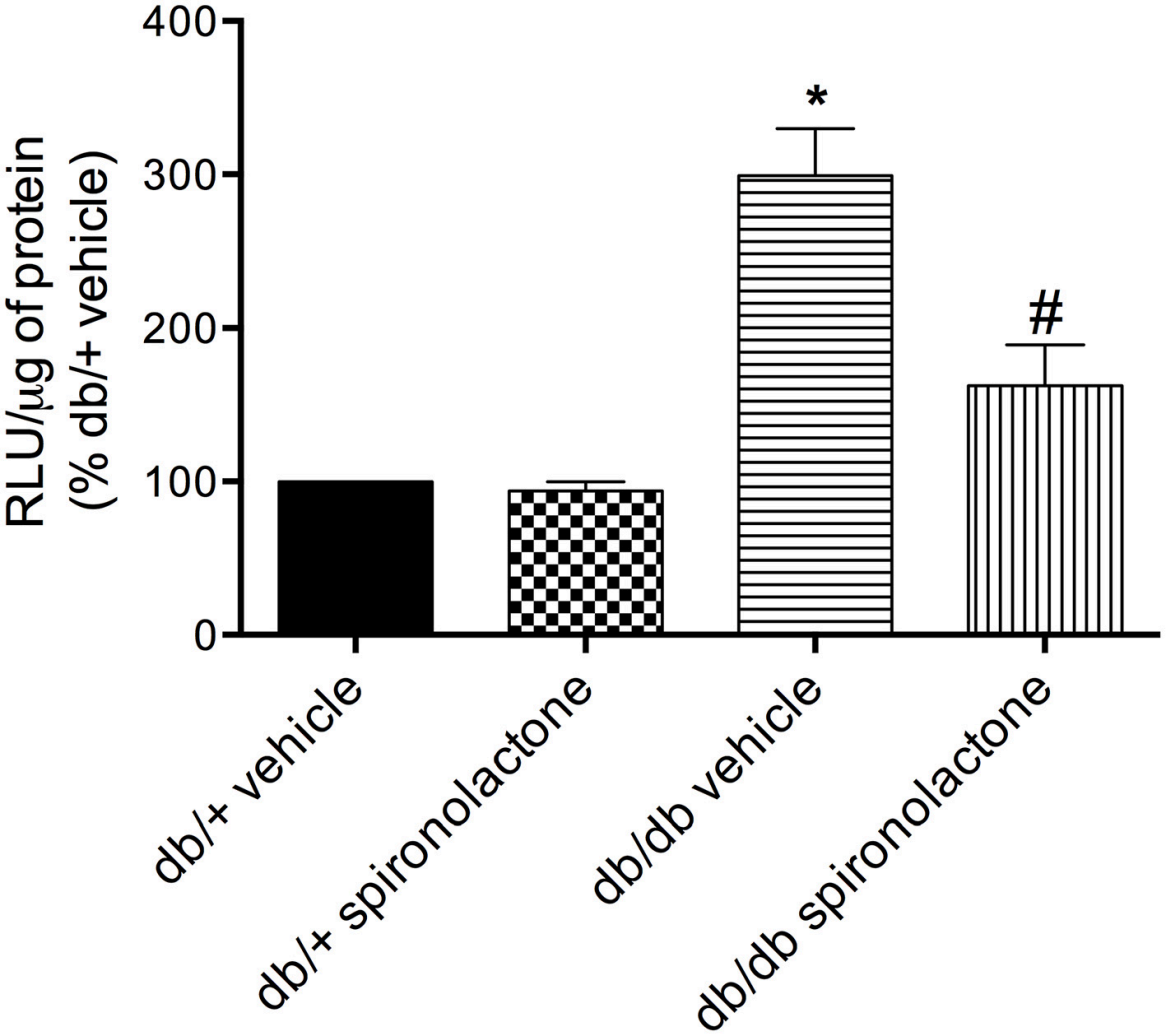
**Lucigenin chemiluminescence and production of ROS in aortas of db/db mice and db/+ treated with spironolactone or vehicle for 6 weeks**. Values are expressed as % of fluorescence respectively to that in arteries from vehicle-treated db/+ mice. Data are expressed as mean ± SEM. ^*^P < 0.05 vs. respective db/+ group, ^#^*P* < 0.05 vs. db/db vehicle; RLU, Relative Light Units; *n* = 6.

**Figure 7:**
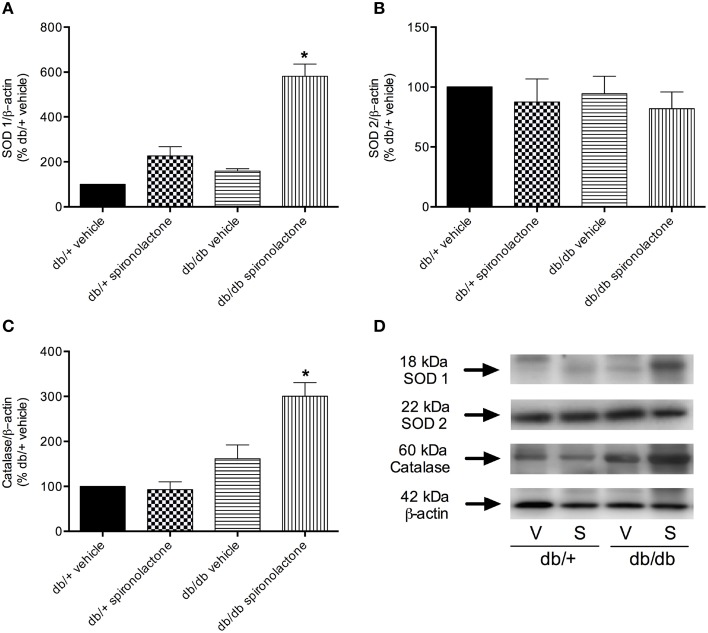
**Protein expression of SOD1 (A), SOD2 (B), and catalase (C) in mesenteric arteries from db/db and db/+ mice treated with spironolactone or vehicle for 6 weeks**. Representative images are displayed in **(D)**. β-actin expression was also determined and used as an internal control. Data are expressed as mean ± SEM. ^*^P < 0.05 vs. all other groups; V, vehicle; S, spironolactone, *n* = 5.

The antioxidant potential of the MR antagonist was evaluated in the vasculature of db/db and db/+ mice. NADPH-dependent superoxide anion generation was measured in aortic homogenates from db/+ and db/db mice. Figure 6 shows that lucigenin-derived luminescence was significantly increased in arteries from db/db mice compared to db/+ arteries. Spironolactone reduced superoxide anion generation in db/db mice. Although expression of the antioxidant enzymes SOD1, SOD2, and catalase were similar in mesenteric arteries from db/db and db/+ mice, spironolactone treatment enhanced vascular SOD1 and catalase expression in db/db mice (Figure 7).

## Discussion

The present study demonstrates that vascular dysfunction in db/db mice is attenuated by MR antagonism through processes associated with reduced vascular oxidative stress and improvement of NO/sGC-mediated responses. Our data provide additional information on mechanisms whereby MR antagonists, such as spironolactone, protect against diabetes-associated vasculopathy and further support the idea that MR antagonists have beneficial vascular effects in diabetes-associated conditions.

Obesity and DM2 are closely related with high aldosterone levels, and MR antagonist therapy has shown positive effects on cardiovascular complications associated with these diseases (Briones et al., 2012; Silva et al., 2015). Spironolactone treatment produces beneficial cardiovascular effects independently of a reduction of aldosterone levels (Mejía-Vilet et al., 2007; Resch et al., 2011). In line with these findings we found that MR antagonist therapy did not change aldosterone levels in the experimental groups, including diabetic mice, which still presented higher levels of aldosterone. Aldosterone plays a positive role to enhance insulin levels, as well as to induce insulin resistance (Patel and Mehta, 2012; Bruder-Nascimento et al., 2014). Rats treated with aldosterone present insulin resistance, by mechanisms that involve MR receptor activation and oxidative stress (Patel and Mehta, 2012; Sherajee et al., 2012). We have recently shown that db/db mice treated with the MR antagonist spironolactone have improved metabolic and cardiovascular profile (Silva et al., 2015). In the present study, spironolactone treatment reduced, but not normalized insulin levels in db/db mice, which exhibit increased plasma insulin levels. Although db/db mice are obese and diabetic, they do not develop high blood pressure. Spironolactone treatment did not influence blood pressure, in line with previous reports (Briones et al., 2012; Bruder-Nascimento et al., 2015; Silva et al., 2015).

MR antagonists inhibit Na^+^ reabsorption in the distal tubule, increasing the urinary excretion of Na^+^ while retaining K^+^ (Epstein and Calhoun, 2011). We found no differences in the Na^+^ content among the groups, but spironolactone treatment increased plasma K^+^ levels in both db/+ and db/db mice, indicating that the treatment was effective.

Cardiovascular disease and nephropathy, due in large part to vascular injury, are the main morbidities associated with diabetes (Global Burden of Metabolic Risk Factors for Chronic Diseases, 2014; Garg et al., 2015). Vascular dysfunction has been consistently reported in diabetic patients and in experimental models of type 1 and type 2 diabetes (Wenzel et al., 2008; Lau et al., 2013; Chen et al., 2014; Bruder-Nascimento et al., 2015; Garg et al., 2015). Although vascular dysfunction is a well-defined phenomenon in diabetes, the mechanisms involved are still unclear.

Vascular dysfunction in diabetic patients is ameliorated by treatment with MR antagonists, indicating that MR blockade may prevent or revert cardiovascular disease in patients with DM2 (Garg et al., 2015). In arterial hypertension and heart failure, aldosterone has been shown to mediate maladaptive changes in the cardiovascular system by mechanisms that involve Nox activation, oxidative stress, reduced NO bioavailability and ultimately, vascular dysfunction (Zannad, 1995; Virdis et al., 2002; Callera et al., 2005; Calhoun, 2006). In these conditions, MR blockade reduces cardiac inflammation, oxidative stress, fibrosis, remodeling, hypertrophy, and improves cardiac and vascular function (Virdis et al., 2002; Callera et al., 2005, 2011; Brown, 2013; Velmurugan et al., 2013; Zhang et al., 2014).

Findings from our study demonstrate that treatment with a MR antagonist abrogates vascular dysfunction and altered NO/cGMP signaling in DM2 mice. Db/db mice presented vascular dysfunction (reduced endothelium-dependent and endothelium-independent vasodilation, represented by decreased Ach- and SNP-induced relaxation, respectively), oxidative stress, and reduced NO bioavailability, in line with the literature (Liu et al., 2014).

Nitric oxide (NO), a major regulator of vascular tone (Malinski et al., 1993; Hakim et al., 1996; Fels et al., 2010), is synthesized mainly by eNOS in the vascular system. Upon its synthesis in the endothelium, NO diffuses to the adjacent vascular smooth muscle cells (VSMC), where it activates soluble sGC, which, via cGMP, decreases myosin light chain kinase activity and increases Ca^2+^-ATPase activity, thereby inducing vasodilation (Förstermann and Münzel, 2006; Fels et al., 2010).

Aldosterone influences NO bioavailability via both genomic and non-genomic pathways (Fels et al., 2010; Callera et al., 2011; Nguyen Dinh Cat and Jaisser, 2012; Bruder-Nascimento et al., 2014). Short-term exposure to aldosterone seems to increase NO bioavailability (Romagni et al., 2003; Schmidt et al., 2003; Michea et al., 2005; Nietlispach et al., 2007) whereas long-term exposure leads to impaired NO bioavailability and signaling. *In vitro*, prolonged exposure to aldosterone decreases nitrite concentration in the medium, cellular cGMP concentrations, and attenuates ability of rat mesenteric arteries to contract (Virdis et al., 2002; Nagata et al., 2006; Oberleithner et al., 2009). Treatment with MR antagonists abolishes aldosterone-effects, suggesting that MR mediate aldosterone-induced decreased NO bioavailability. Although it is clear that chronic aldosterone exposure leads to impairment of NOS activity, the specific mechanisms underlying this effect are still unclear. Of importance, our data show that MR blockade augmented NO-dependent responses, reinforcing the notion that aldosterone is an important regulator of NO bioavailability in the vascular system (Fels et al., 2010).

Endothelium-dependent Ach-mediated relaxation in arteries from db/db mice was not sensitive to L-NAME effects as observed in the remaining groups. This suggests reduced involvement of NO, and possibly a contribution from another endothelial factor [e.g., a cyclooxygenase-derived or a non-NO and non-prostanoid endothelial factor, such as prostacyclin or an endothelium-dependent hyperpolarizing factor (EDHF)] to Ach responses in arteries from diabetic mice. On the other hand, Ach-induced vasodilation was almost completely abrogated by L-NAME in arteries from db/db mice treated with spironolactone, indicating that treatment with the MR antagonist increased NO bioavailability. Accordingly, spironolactone treatment has been shown to improve endothelial dysfunction and increase NO bioavailability in patients with heart failure (Farquharson and Struthers, 2000). Also, eplerenone increases the production of NO as well as the activity of antioxidant enzymes, such as MnSOD and CuZnSOD, in macrophages from patients with heart failure (Labuzek et al., 2014).

It is well known that there is an up-regulation of EDHF-mediated relaxation in resistance arteries in situations of reduced endothelial NO availability (Waldron et al., 1999; Ding et al., 2000). For example, in eNOS knockout mice, EDHF-mediated responses play a compensatory role in the absence of endothelial NO in agonist- and flow-induced endothelium-dependent vasodilatation as well as in the basal regulation of myogenic tone (Waldron et al., 1999; Brandes et al., 2000; Ding et al., 2000; Huang et al., 2000; Scotland et al., 2005). Experimental evidence indicates that increased EDHF is an important compensatory mechanism for maintaining endothelium-dependent relaxation in type 2 diabetes and dyslipidemia, conditions where endothelium production of NO and prostacyclin are compromised, particularly at early disease stages (Krummen et al., 2005; Wölfle and de Wit, 2005; Beltowski et al., 2006; Pannirselvam et al., 2006). Although we have not directly investigated the contribution of prostanoids and/or EDHF to Ach responses, our data indicate that: NO-mediated relaxation responses to Ach are impaired/decreased in arteries from diabetic mice; another endothelium-derived component mediates responses to Ach in arteries from db/db mice; and spironolactone treatment in db/db mice improves the NO-dependent component of Ach-induced relaxation.

Aldosterone also increases ROS production via activation of NADPH oxidase (Nox) isoforms. Whereas aldosterone administration increases vascular oxidative status, therapy with MR antagonists abrogates oxidative stress (Callera et al., 2005; Zhang et al., 2014), suggesting that aldosterone-induced ROS formation depends on MR receptor. We have recently shown that aortae from db/db mice exhibit enhanced Nox expression and activity (Bruder-Nascimento et al., 2015) and that MR antagonist therapy reduces oxidative stress possibly via modulation of Nox enzymes-dependent mechanisms (Silva et al., 2015).

The present data show that treatment with the MR antagonist spironolactone restored vascular ROS generation to normal levels, reinforcing that MR intervenes on redox balance in diabetic conditions, possibly increasing antioxidant enzymes, as we observed in this study. Accordingly, redox imbalance may be linked to reduced antioxidant enzymes activity/expression. Spironolactone upregulated vascular expression of SOD1 and catalase, indicating that improvement of vascular function is associated with increased antioxidant capacity. Our findings are partially in line with a previous study showing that eplerenone treatment restores antioxidant enzymes in the kidney from hypertensive rats (Onozato et al., 2007). Although we have no mechanistic explanation for the differential effects of spironolactone on SOD1 and catalase expression in control and db/db mice, it is possible that MR blockade increases antioxidant enzymes only in db/db mice to counterbalance ROS overproduction, since the antioxidant enzymes content in the vasculature from control mice was enough keep normal oxidant status.

ROS directly interferes with NO production and signaling by different mechanisms. Superoxide anion (O${}_{2}^{-}$) reacts avidly with vascular NO to form ONOO^−^, thus accelerating degradation of NO. This has been reported in different cardiovascular and metabolic diseases, including DM2 (Touyz, 2004). Our data show increased eNOS activity, inferred by eNOS phosphorylation in Ser^1177^, in the vasculature of diabetic mice. Normal eNOS function requires dimerization of the enzyme, the presence of the substrate L-arginine, and the essential cofactor (6R)-5,6,7,8-tetrahydro-L-biopterin (BH_4_). The cofactor BH_4_ is highly sensitive to oxidation by ONOO^−^. Diminished levels of BH_4_ promote O${}_{2}^{-}$ production by eNOS, a phenomenon known as eNOS uncoupling. Although, we have not determined eNOS uncoupling or ONOO^−^ levels, we cannot exclude the possibility that MR antagonist-associated vasoprotection occurs through improvement of NO formation via a decrease in eNOS uncoupling (Förstermann et al., 1994; Förstermann and Münzel, 2006).

NO induces vasodilation by stimulating sGC and increasing cGMP in VSMC (Förstermann et al., 1994; Förstermann and Münzel, 2006; Fels et al., 2010). Mesenteric arteries from db/db mice also exhibited attenuated relaxation to SNP, a NO-donor, and to the sGC stimulator, BAY 41-2272, which indicate that vascular dysfunction observed in diabetic mice also relies on altered sGC signaling. In addition, reduced protein content of sGC β subunit was detected in the vasculature of db/db mice. sGC is a ROS-sensitive protein, and increased oxidative stress affects the heme-containing NO-binding site of the enzyme by decreasing its expression and impairing NO-induced activation, resulting in less effective responses to NO donors (Stasch et al., 2011). Oxidation of the heme group on sGC leading to its dissociation from the enzyme and the generation of NO-insensitive sGC has been reported in experimental models of hypertension and hyperlipidemia, as well as in patients with cardiovascular diseases and DM2 (Gladwin, 2006; Stasch et al., 2006, 2011). Spironolactone treatment restored endothelium-independent SNP-induced vasorelaxation, an event associated with upregulation of sGC. In addition, tempol, an antioxidant agent, abolished altered responses to SNP, further strengthening the possibility of sGC oxidation. Since 8-Br-cGMP-induced relaxation was not altered in arteries from diabetic mice, it seems that the sensitivity of VSMC to the effects of cGMP are preserved in diabetic conditions.

It is interesting that tempol had no effects on endothelium-dependent relaxation, but abrogated decreased endothelium-independent relaxation in arteries from db/db mice. As previously mentioned, sGC plays a crucial role to mediate NO-induced relaxation, by increasing cGMP in VSMC. sGC can be oxidized, which leads to impaired vascular relaxation. An antioxidant agent, like tempol, might blunt sGC oxidation and then revert its decreased activity. As Ach-induced relaxation relies on a non-NO component in arteries from db/db mice, it is possible that sGC was not involved in the relaxation responses and, therefore, the effects of tempol were insignificant at this condition. Spironolactone treatment increased NO bioavailability and reduced ROS generation. In this condition (of increased NO bioavailability and reduced ROS), sGC might be functional again and able to respond to NO effects. In addition, our findings with BAY 41-2272 indicate that impaired vascular relaxation in diabetic mice is associated with impaired sGC activation. Spironolactone restored BAY 41-2272-induced relaxation, possibly by decreasing sGC oxidation and/or increasing its protein expression. A limitation of our study includes the absence of protocols investigating oxidation of sCG in arteries from vehicle- and spironolactone-treated diabetic mice.

In conclusion, our data demonstrate that spironolactone, a MR antagonist, improves vascular function by reducing oxidative stress, upregulating antioxidant systems and improving NO/sGC signaling. These data imply a role for aldosterone in vascular dysfunction and suggest that spironolactone may have vasoprotective effects in diabetes-associated cardiovascular complications.

## Author contributions

All authors contributed to the work presented in this paper. MS designed and performed all experiments, analyzed, interpreted and organized the data and wrote the paper. TB, SC, RL, RF, and FM designed and performed specific experiments and helped in the interpretation of results. RT and RT coordinated and mentored the project execution. All authors approved the paper for publication.

### Conflict of interest statement

The authors declare that the research was conducted in the absence of any commercial or financial relationships that could be construed as a potential conflict of interest.
